# Quantifying the impact of social groups and vaccination on inequalities in infectious diseases using a mathematical model

**DOI:** 10.1186/s12916-018-1152-1

**Published:** 2018-09-26

**Authors:** James D. Munday, Albert Jan van Hoek, W. John Edmunds, Katherine E. Atkins

**Affiliations:** 1 Centre for Mathematical Modelling of Infectious Diseases, London School of Hygiene & Tropical Medicine, London, UK; 2 Department of Infectious Disease Epidemiology, Faculty of Epidemiology and Population Health, London School of Hygiene & Tropical Medicine, London, UK; 3 National Institute for Public Health and the Environment (RIVM), Bilthoven, The Netherlands; 4 Centre for Global Health, Usher Institute of Population Health Sciences and Informatics, Edinburgh Medical School, The University of Edinburgh, Edinburgh, UK

**Keywords:** Inequality, Mathematical modelling, Influenza, Rubella, Social groups, Vaccination

## Abstract

**Background:**

Social and cultural disparities in infectious disease burden are caused by systematic differences between communities. Some differences have a direct and proportional impact on disease burden, such as health-seeking behaviour and severity of infection. Other differences—such as contact rates and susceptibility—affect the risk of transmission, where the impact on disease burden is indirect and remains unclear. Furthermore, the concomitant impact of vaccination on such inequalities is not well understood.

**Methods:**

To quantify the role of differences in transmission on inequalities and the subsequent impact of vaccination, we developed a novel mathematical framework that integrates a mechanistic model of disease transmission with a demographic model of social structure, calibrated to epidemiologic and empirical social contact data.

**Results:**

Our model suggests realistic differences in two key factors contributing to the rates of transmission—contact rate and susceptibility—between two social groups can lead to twice the risk of infection in the high-risk population group relative to the low-risk population group. The more isolated the high-risk group, the greater this disease inequality. Vaccination amplified this inequality further: equal vaccine uptake across the two population groups led to up to seven times the risk of infection in the high-risk group. To mitigate these inequalities, the high-risk population group would require disproportionately high vaccination uptake.

**Conclusion:**

Our results suggest that differences in contact rate and susceptibility can play an important role in explaining observed inequalities in infectious diseases. Importantly, we demonstrate that, contrary to social policy intentions, promoting an equal vaccine uptake across population groups may magnify inequalities in infectious disease risk.

**Electronic supplementary material:**

The online version of this article (10.1186/s12916-018-1152-1) contains supplementary material, which is available to authorized users.

## Background

Reductions in global infectious disease burden have uncovered inequalities in infectious disease health outcomes [1–7]. These inequalities often reflect a disproportionately high incidence observed amongst the most deprived and vulnerable in society [4, 8–10]. Implementing equitable public health care relies on prioritising effective interventions that control the drivers of these inequalities [11].

There may be many contributing factors to inequalities in reported infectious disease health outcomes. Some of these factors have a direct and proportional impact on the relative reported disease burden between social groups, for example, the severity of disease experienced [12, 13], the propensity to seek health care [14] and the reporting rate of disease [15]. In contrast, other factors affect the transmission of infection and may result in non-linear changes in the relative disease burden between social groups. This latter group of factors include differences in social contact, both within and between social groups, and differences in the susceptibility to infection and infectiousness.

Although indistinguishable when their effects are measured using reported disease burden, these drivers have different implications for delivering equitable public health interventions. For example, in the 2009 H1N1 pandemic influenza A (pH1N1) disparities in health outcomes between social groups were identified globally. In particular, British Pakistanis had a 3.4 times increased risk of mortality relative to the White British population [16]; many ethnic minority groups (Black, South Asian and Southeast Asian) had a higher risk (odds ratio (OR) of 1.33–4.5) of exposure than white Canadians in Ontario [17]; Pacific populations were twice as likely to be exposed to infection than the rest of the New Zealand population [18]. Although these examples would likely present as increased clinical burden in particular sub-groups, the drivers of these differences are difficult to determine. Even though the results from New Zealand indicate differences in transmission rate between sub-groups, the seroprevalence data do not provide enough information to identify the specific driver responsible.

Vaccination is an important intervention in infectious disease control because it reduces disease burden in those vaccinated as well as reducing onward transmission to unvaccinated people. The strength of this indirect protection non-linearly depends on the transmission rate [19]. Therefore, if inequalities are caused by differences in transmission between social groups, vaccination may benefit some groups more than others. The impact of vaccination on inequality in infectious disease outcome is therefore unclear.

To address this gap in our knowledge, we developed a novel mathematical model of the transmission of two vaccine-preventable infections circulating in a population with two social groups characterised by different transmission properties. To quantify the effect of differences in transmission on disease inequality between the social groups, we parameterised the model using realistic estimates of susceptibility and contact structure informed by empirical social mixing data. Using our model, we investigated how the overall impact of vaccination is distributed between two social sub-groups, as well as the effect on inequality in disease incidence.

In addition, we determined the optimal vaccine allocation needed to eliminate inequality.

## Methods

**Fig. 1:**
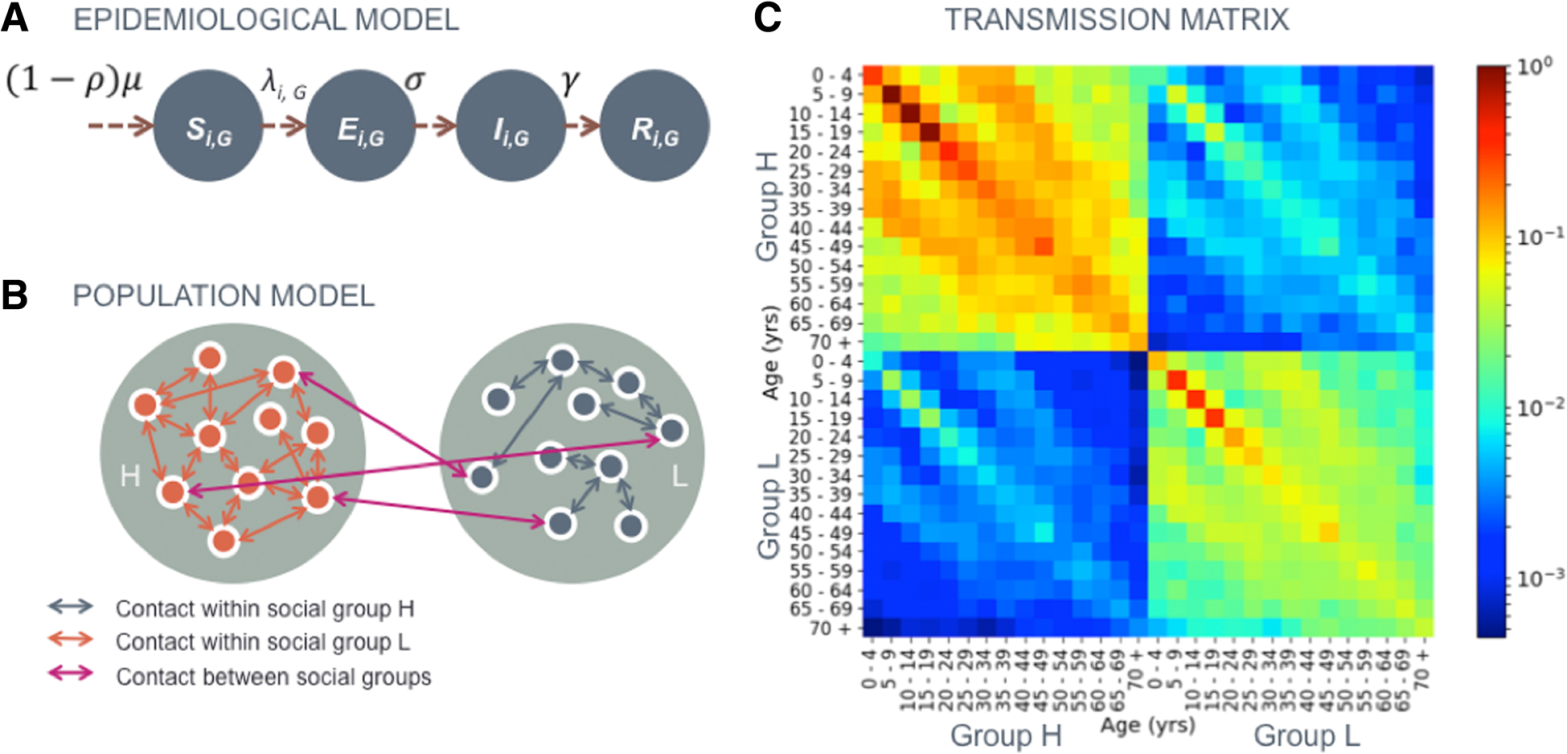
Summary of the mathematical model used to quantify inequalities between social groups *H* (high risk) and *L* (low risk). **a** The epidemiological model, where ***S***_***i,G***_***, E***_***i,G***_***, I***_***i,G***_***. R***_***i,G***_ and ***λ***_***i,G***_ are the proportion susceptible, infected but not infectious, infectious, recovered and force of infection in age group *i* and social group *G* (either group *H* or group *L*), ***ρ*** is the proportion vaccinated, ***σ*** is the rate at which infected individuals become infectious and ***γ*** is the rate of recovery from infection. Population also moves out of these groups into other age groups and are removed when they die (not shown in this schematic). **b** A schematic of the population model with higher contact rate in group *H* than group L; the groups also differ in susceptibility (not shown). **c** An example transmission matrix, showing the relative transmission rate between age and social groups with all social mixing and susceptibility assumptions included with parameterisation ***χ =*** **0.6*, η =*** **0.6*, ξ =*** **0.05** (rates normalised such that the highest transmission group 10–14 years old in group *H* has a rate of 1***.*** The same age group has a rate of 0.36 within group *L* (low susceptibility and reduced contact rate, ***χ*** and ***η***), 0.05 from group *L* to group *H* (between-group contact rate, ***ξ***) and 0.03 from group *H* to group *L* (between-group contact rate and reduced susceptibility, ***ξ*** and ***η***)

We developed a novel mathematical model to evaluate whether differences in contact rate and the susceptibility to infection between two social groups can explain disease inequality across a population. We used this model to quantify how a vaccination programme affects these inequalities. Our mathematical model combined a dynamic epidemiological model of disease transmission with an age-structured population model of two distinct social groups (Fig. 1).

### Population model

To simulate the demographics of a high-income country, we modelled a stable age distribution with birth rate equal to death rate, a life expectancy of 80 years and mortality only occurring after 70 years of age at a constant rate. The population model was stratified into *n*_*age*_ = 15 age groups (0–4, 5–9, …, 65–69, 70+ years) with continuous ageing between age groups. The age-structured population model was further stratified into two social groups of equal size, with the same proportion of male and female and an identical age structure. Throughout the paper, the social groups with high and low transmission are labelled group *H* and group *L*, respectively.

### Epidemiological model

Our dynamic transmission model tracked the proportion of the population as susceptible (*S*), infected but not infectious (*E*), infectious (*I*) and permanently immune to infection (*R*) (Fig. 1a).

The transmission between and within the two social groups was captured by three mechanisms. The first two control the underlying differences between the two social groups that are potential drivers of inequality: (1) a difference in contact intensity between the two groups, expressed as the relative rate at which members of group *L* interact with members of their own group, compared to the rate at which members within group *H* interact with one another (‘contact intensity’, 0 < *χ* < 1), and (2) a difference in susceptibility to infection, expressed as the relative susceptibility for members of group *L* compared to members of group *H* (‘susceptibility’, 0 < *η* < 1). The third mechanism determines the integration of the two social groups: (3) the relative rate at which individuals from one social group contact members of the opposite social group (‘integration’, 0 < *ξ* < 1). For example, *ξ* = 0.15 corresponds to contact between group *H* and group *L* at 15% of the rate of contact within group *H*. The rate of contact between the groups remained symmetrical; i.e. the rate of contact from group *H* to group *L* was the same as the rate of contact from group *L* to group *H*. The force of infection, *λ*, for the susceptible population in age group *i* and social group *H* or *L* is therefore dependent on the social group-specific susceptibility, the age- and social group-specific contact rate and the reproductive number, *R*_0_, of the disease (Fig. 1c) and can be expressed as:

1 $$ {\lambda}_{i,H}=\sum \limits_{j=1}^{15}{r\beta}_{ij}\left({I}_{j,H}+{\xi I}_{j,L}\right) $$

2 $$ {\lambda}_{i,L}=\sum \limits_{j=1}^{15}{r\eta \beta}_{ij}\left({\xi I}_{j,H}+{\chi I}_{j,L}\right) $$

where *β*_*ij*_ is the age-specific transmission rate from age group *j* to age group *i*, and *I*_*j*, *H*_ and *I*_*j*, *L*_ are the proportion infectious in age group *j* and social groups *H* and *L*, respectively.

To keep *R*_0_ constant when the relative contact rate (*χ*), susceptibility (*η*) and integration (*ξ*) of the social groups were changed, we scaled the force of infection using a linear operator, *r*. This approach allows parameters of interest (relative contact rate (*χ*), susceptibility (*η*), integration (*ξ*) and *R*_0_) to be varied independently from each other (Additional file 1). All modelling and analysis was performed using Python 2.7.12 [20].

### Parameterisation

#### Disease scenarios

We parameterised our model for two vaccine-preventable diseases: seasonal influenza and rubella. We quantified the incidence in the total population for both diseases. For influenza, we also quantified the incidence in those aged 60 years and over, who are at risk for severe complications following infection. For rubella, we quantified the incidence in women of childbearing age (WCA) (15–45 years), who serve as a proxy for children born with congenital rubella syndrome after their mothers become infected during pregnancy. The reproduction number, incubation period and infectious period for both diseases were parameterised from the literature (Table 1). The contact rate between age groups was parameterised with empirical social mixing data collected in the UK arm of the POLYMOD contact survey [21].

**Table 1:** Model parameter values used in base case and sensitivity analyses

	Symbol	Primary analysis	Sobol range^b^
Population parameters			
Difference in transmission (either):^a^			
Within-group mixing (’contact’)	*χ*	0.65–0.95	0.65–1.54
Relative susceptibility of group *L* to group *H* (’susceptibility’)	*η*	0.65–0.95	0.65–1.54
Quantity of out-group mixing relative to within-group mixing of group *H* (’integration’)	*ξ*	0.05–0.25	0.05–0.25
Relative vaccine uptake in group *H* to group *L*	*V*_*H*_/*V*_*L*_	1.0	0.70–1.43
Epidemiological parameters			
Basic reproduction number [48, 49]	*R*_0_		
Influenza		1.8	1.5–4.0
Rubella		6.5	5.0–8.0
Pre-infectious period (days) [50]	*σ*		
Influenza		2.6	2.6
Rubella		14.0	14.0
Infectious period (days) [50]	*γ*		
Influenza		4.0	4.0
Rubella		11.0	11.0

^a^One parameter value set to 1.0 whilst the other adjusted over the ‘primary analysis range’

^b^Ranges were set so the mid value is the ‘base case’, which was 1.0 (no difference) for factors which vary for group *L* relative to group *H*

#### Inequality mechanisms

##### Integration

We informed the parameterisation of *ξ*, the rate of contact between social groups, relative to the rate of contact within group *H*, using social contact data from the UK arm of the POLYMOD study [21]. We assumed that all household contacts were within their own social group, with a further 70–90% of non-household contacts also within their own social group. The relative rate of contact between social groups, *ξ*, was estimated as 0.05–0.25 (Additional file 1).

##### Relative contact rate

The feasible range for the contact intensity parameter, *χ*, the relative rate contact within group *L* compared to group *H*, was also informed by the POLYMOD contact data. For each of the 15 age groups we sorted the participants into quintiles by their household size. We then recombined the age groups, quintile by quintile, to recover five equally sized groups. For each participant, we calculated the total number of contacts from within each person’s own social group (using the same assumption as above that all household contacts and 70–90% of non-household contacts were with members of their own social group). The contact intensity parameter *χ* was then estimated by evaluating the ratio of the total number of within-group contacts for individuals in every unique pair of quintiles. We estimated the range of ratios as 0.65–0.95 (Additional file 1).

##### Relative susceptibility

Given the disease-specific consideration regarding previous exposure to obtain a parameter for the relative susceptibility (*ξ*), we investigated the same range of 65–95% susceptibility in group *L* compared to group *H*.

### Primary analysis: quantifying inequalities

The inequality in the population was expressed by the relative risk of infection in the high mixing group (group *H*) relative to the low mixing group (group *L*). We calculated this relative risk across the overall population and for the disease-specific risk groups. For influenza, we calculated the cumulative relative risk over the course of a single outbreak. For rubella, we measured the relative annual infection risk at endemic equilibrium to ensure that both rate of transmission and age-specific prior exposure to infection were accounted for in our calculation.

### Vaccination

For both diseases, we assumed that a proportion of individuals become immunised after vaccination—an ‘effective coverage’. Consistent with disease-specific immunity profiles, we assumed no waning of vaccine protection over the period of evaluation (lifetime for rubella or one influenza season). Effective coverage for influenza vaccination was identical across all age groups from the beginning of the season; for rubella, vaccine was administered at birth. To allow comparison of results between the two diseases with different *R*_0_ values, we express the effective vaccine coverage as a fraction of the critical vaccination threshold (CVT), 1 – 1/*R*_0_, i.e. the minimum proportion of the population required to be vaccinated to interrupt transmission. We evaluated the relative risks of infection with no vaccination and with vaccination at 80% of the CVT. Unless otherwise stated, the effective coverage was assumed to be identical between social groups.

### Identifying the drivers of inequality

To evaluate the relative importance of the model parameters as drivers for inequality, we used a variance-based global sensitivity analysis, the total Sobol′ sensitivity index (*S*_*T*_) [22, 23], which calculates the proportion of the variance in the relative risk attributable to each parameter and combinations thereof.

## Results

### Underlying epidemiology

**Fig. 2:**
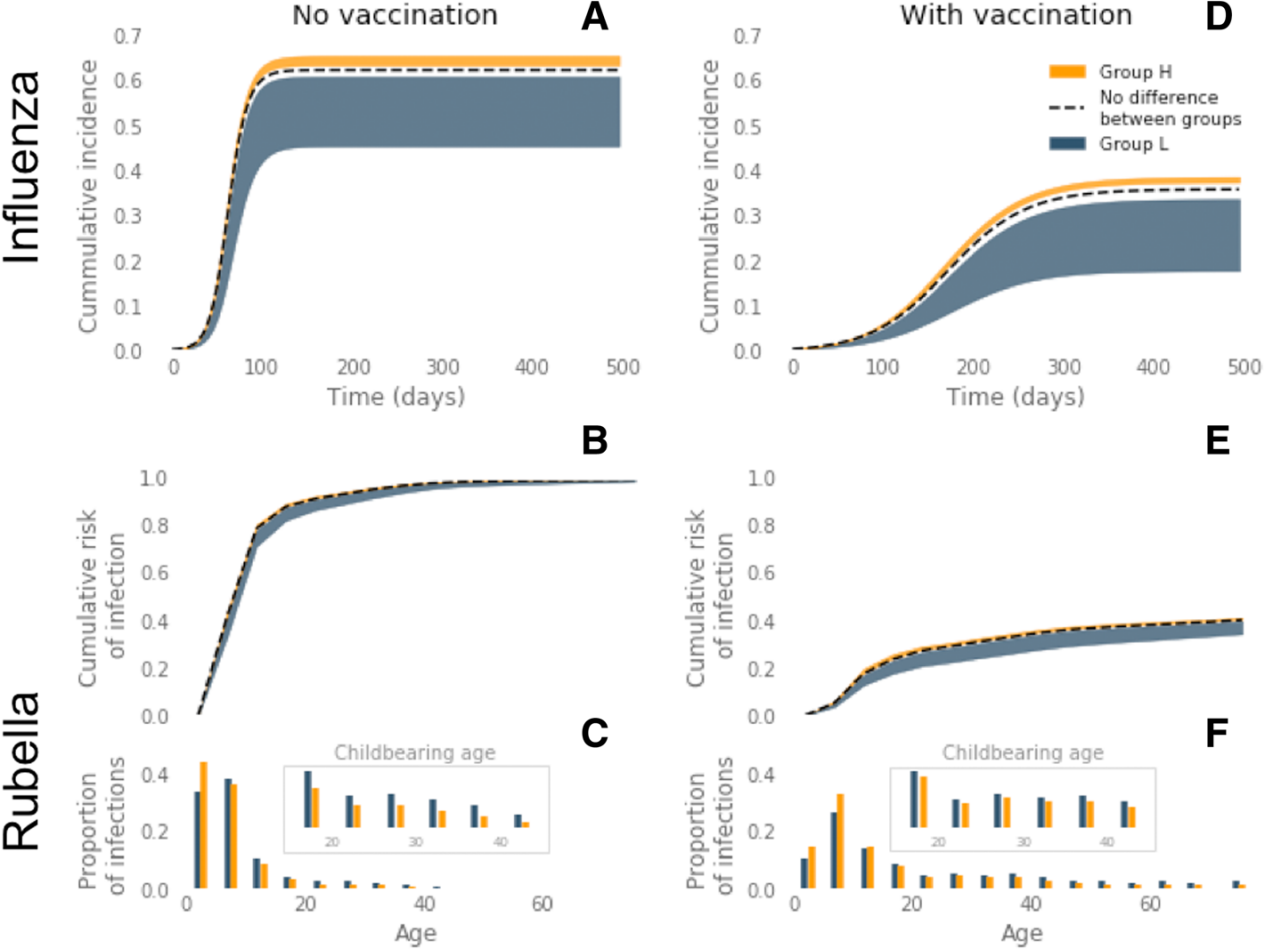
Epidemiology predicted by the mathematical model for seasonal influenza and rubella with no differences between two population groups (*black dashed line*) and with differences in susceptibility and contact rate for group *H* (*orange region*) and group *L* (*navy region*) across feasible range of contact rate within social groups (*χ* = 0.65 – 0.95) and base case values of integration (*ξ* = 0.15) and susceptibility (Table 1). **a** Cumulative incidence of influenza over a single outbreak with no vaccination. **b** Proportion of population infected with rubella by age at endemic equilibrium with no vaccination. **c** Proportion of all infections acquired in each 5-year age group, with no vaccination. **d** Cumulative incidence of influenza with 37% vaccine uptake (80% of the critical vaccination threshold (CVT)). **e** Proportion of population infected with rubella by age with 67% vaccine uptake (80% of the CVT). **f** Proportion of all infections acquired in each 5-year age group, with 67% vaccine uptake (80% of the CVT)

We ran simulations with no vaccination and no epidemiological differences between group *H* and group *L* (i.e. setting *χ*, *η*, *ξ* = 1). We found that the influenza epidemic lasted approximately 21 weeks with a cumulative attack rate of 62% across all age groups and 40% amongst those older than 60 years. For rubella at endemic equilibrium 99.4% of the population were infected before death (95% before the age of 30 years), and the mean age of infection was 8 years. The annual incidence amongst WCA was 66 per 100,000 (Fig. 2).

### Pre-vaccination inequalities

#### Influenza

**Fig. 3:**
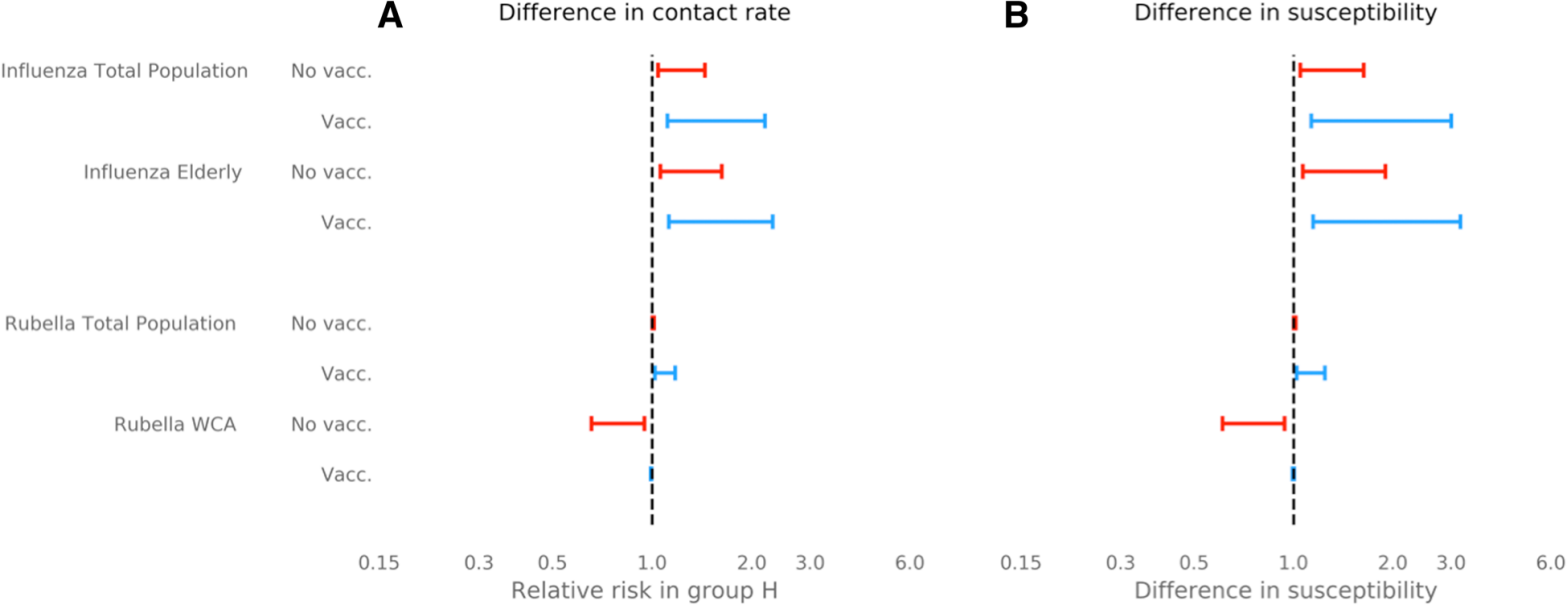
Risk of infection in group *H* relative to group *L* in the total population and in risk groups, elderly and women of childbearing age (*WCA*). Relative risks shown with no vaccination and vaccination at 80% of critical vaccination threshold (37% for influenza and 67% for rubella). Forest plots show ranges of relative risk for fixed integration of *ξ* = 0.15 and a range of **a** ratio of in-contact rate in social groups (*χ* = 0.65–0.95) and **b** ratio of susceptibility in social groups (*η* = 0.65–0.95)

**Fig. 4:**
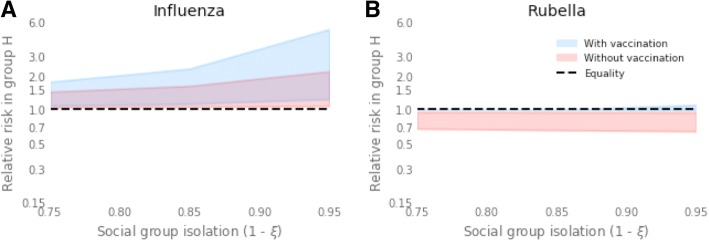
Full range of relative risk in **a** influenza in the elderly (60+ years) and **b** rubella in women of childbearing age (15–45 years), due to differences in contact rate (***χ=*** 0.6–0.9) as isolation between sub-groups varies (***ξ*** = 0.05–0.15). *Red shaded region* shows range of relative risk with no vaccination, *blue shaded region* shows relative risk with vaccination at 80% of the critical vaccination threshold (37% coverage for influenza, 67% coverage for rubella)

Without vaccination, introducing a relative contact rate of 0.65–0.95 within group *L* compared to group *H* led to a change in cumulative attack rate in both social groups, and hence a change in the relative risk of infection between the two groups (Fig. 2). In particular, across base case values of susceptibility and integration, group *H* experienced a relative risk of infection 1.04–1.44 compared to group *L* (Fig. 3a). This relative risk increased to 1.06–1.62 (an increase of 1–12%) amongst the elderly in group *H*. Less integration between the two groups exacerbated this inequality; when contact between groups was decreased by 67% compared to the base case scenario (*ξ*= 0.05), the relative risk for group *H* increased to 1.06–1.84 (Fig. 4a).

Reducing the susceptibility in group *L* by a factor of 0.65–0.95 relative to group *H*, whilst maintaining base case values of within-group contact and between group integration, led to 1.05–1.63 times more infections in group *H* than group *L* over the course of the outbreak (Fig. 3b). Again, the relative risk amongst the elderly in group *H* was higher than that of the social group as a whole, with a relative risk of 1.05–1.63 under base case assumptions of integration. Relative risk of infection in group *H* increased when the social groups were less integrated relative to the base case scenario to 1.08–2.04 (*ξ* = 0.05) and up to 2.49 in the elderly.

#### Rubella

Unlike our influenza model results, differences in contact rate and susceptibility between the social groups did not result in an inequality in the risk of rubella infection in the whole population (Fig. 3). However, a more intense contact rate in group *H* or a lower susceptibility in group *L* led to a lower age of infection in group *H* relative to group *L* (Fig. 2). This difference in the age of infection resulted in a relative risk of infection for WCA in group *H* of 0.64–0.95 across feasible ranges of both within-group contact rates and susceptibility. In contrast to the influenza risk group, therefore, our model suggests there is an elevated risk for the low-transmission social group (Fig. 3). Again, in contrast to the influenza model results, varying the level of integration between social groups only marginally affected the relative risk of infection across WCA (Fig. 4b).

### Post-vaccination inequalities

#### Influenza

Vaccination with a 37% uptake (80% of the CVT) reduced the cumulative attack rate of seasonal influenza from 62% to 30% when transmission in the social groups was identical (Fig. 2d). However, with differences in contact rate and susceptibility between the two social groups, introducing vaccination increased the inequality between the social groups (Fig. 3). For example the relative risk of 1.04–1.84 before vaccination increased to 1.11–2.18 after vaccination with differences in contact rate, and for differences in susceptibility relative risk increased from 1.05–2.04 before vaccination to 1.13–3.00 after vaccination (with base case integration, *ξ* = 0.15).

Consistent with the results without vaccination, relative risk of infection for group *H* increased when the two social groups were less integrated (Fig. 4a). When the inequality was driven by feasible changes in either within-group contact rate or susceptibility to infection, the relative risk across the whole of group *H* reached 4.83 and 6.99, respectively, when integration was at its lowest value (*ξ* = 0.05). Therefore, vaccination increased the inequality of disease risk in the social group most at risk of infection by 5–241% (Table 2).

**Table 2:** Percentage increase in risk of infection in group *H* relative to group *L* due to vaccination

Driver of inequality	Infection	Population group	Increase in relative risk
Difference in contact rate	Influenza	All	4–162%
		Elderly	3–137%
	Rubella	All	2–39%
		WCA	4–72%
Difference in susceptibility	Influenza	All	5–241%
		Elderly	5–203%
	Rubella	All	2–49%
		WCA	5–86%

Percentage increases in relative risk of infection for the total population (all), women of childbearing age (WCA) and elderly. Results calculated when either the relative within-group contact rate of the two social groups is varied (‘contact’ parameter) or when the relative susceptibility of group *L* to group *H* is varied (‘susceptibility’ parameter) (Table 1). Integration between the social groups is set at its base case value

Although the percentage increase in relative risk after vaccination was less amongst the elderly in group *H* (5–203%), the relative risk remained higher than in the total population, with a maximum relative risk of 5.19 and 7.52 for differences in contact rate and susceptibility, respectively (Fig. 4a).

The marked increase in inequality in risk of influenza infection as a result of vaccination corresponds to the social group *H* benefiting substantially less from the vaccination programme than group *L*.

Sensitivity analysis shows robustness of these results to variation in the relative size and community structure of group *L* and group *H* (Additional file 1: Figure S15).

#### Rubella

An effective vaccination uptake of 67% (80% of the CVT) greatly reduced lifetime risk of rubella in both social groups, with less than 40% of the unvaccinated population experiencing infection over their lifetime (Fig. 2d). With differences in contact rate between the social groups, vaccination caused an inequality to emerge. Specifically, the relative risk of infection in group *H* relative to group *L* increased from 1.01–1.02 to 1.02–1.42, across a feasible range of within-group mixing patterns (Fig. 3a). The same result was found as a consequence of susceptibility differences (Fig. 3b).

Furthermore, vaccination reduced the difference in the age of infection between the two social groups (Fig. 2). The combination of changes in relative risk of infection before death and in age at infection caused a switch in the group most at risk for infection in WCA. Before vaccination the highest relative risk was amongst women in group *L*, whereas with vaccination the WCA in group *H* tended to have a higher risk, with relative risk ranging from 0.99 to 1.16.

Sensitivity analysis shows robustness of these results to variation in the relative size and community structure of group *L* and group *H* (Additional file 1: Figure S16).

### Vaccinating to prevent inequality

**Fig. 5:**
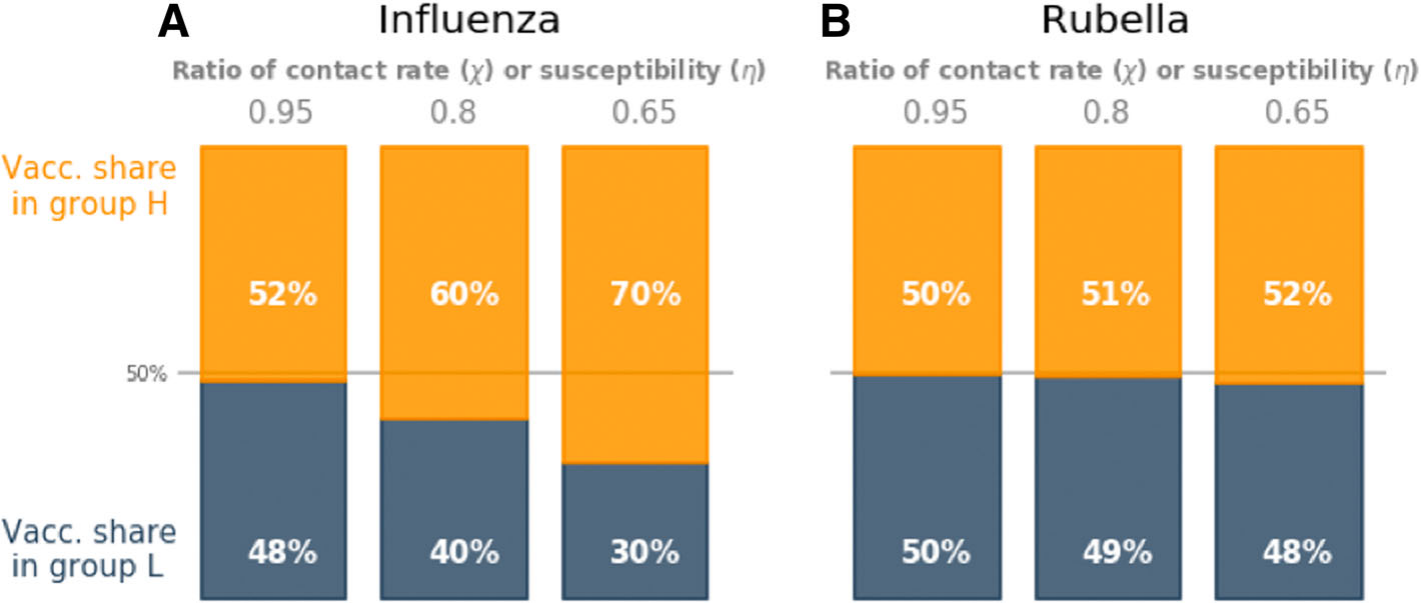
Optimal vaccine allocation between social groups required to control disease inequalities in **a** influenza and **b** rubella. Results shown for ratio of contact rate in social groups (*χ* = 0.65–0.95) and ratio of susceptibility in social groups (*η* = 0.65–0.95). The total vaccination coverage is 80% of the critical vaccination threshold (37% vaccine uptake for influenza, 67% vaccine uptake for rubella)

By increasing the vaccine uptake in group *H* relative to group *L*, the inequalities driven by vaccination, differences in contact rate and differences in susceptibility can be mitigated. To achieve equality in risk of infection for influenza across the entire population, group *H* had to receive 52–70% of the total number of vaccine doses across the feasible ranges of population parameters (Fig. 5a). In contrast, small changes in vaccine dose allocation were required to curb inequality in rubella (50.3–52.3%) (Fig. 5b). The level of integration between the two social groups did not affect the vaccine uptake required in each group to eliminate inequality (results not shown).

### Ranking drivers of inequalities

#### Pre-vaccination era

**Fig. 6:**
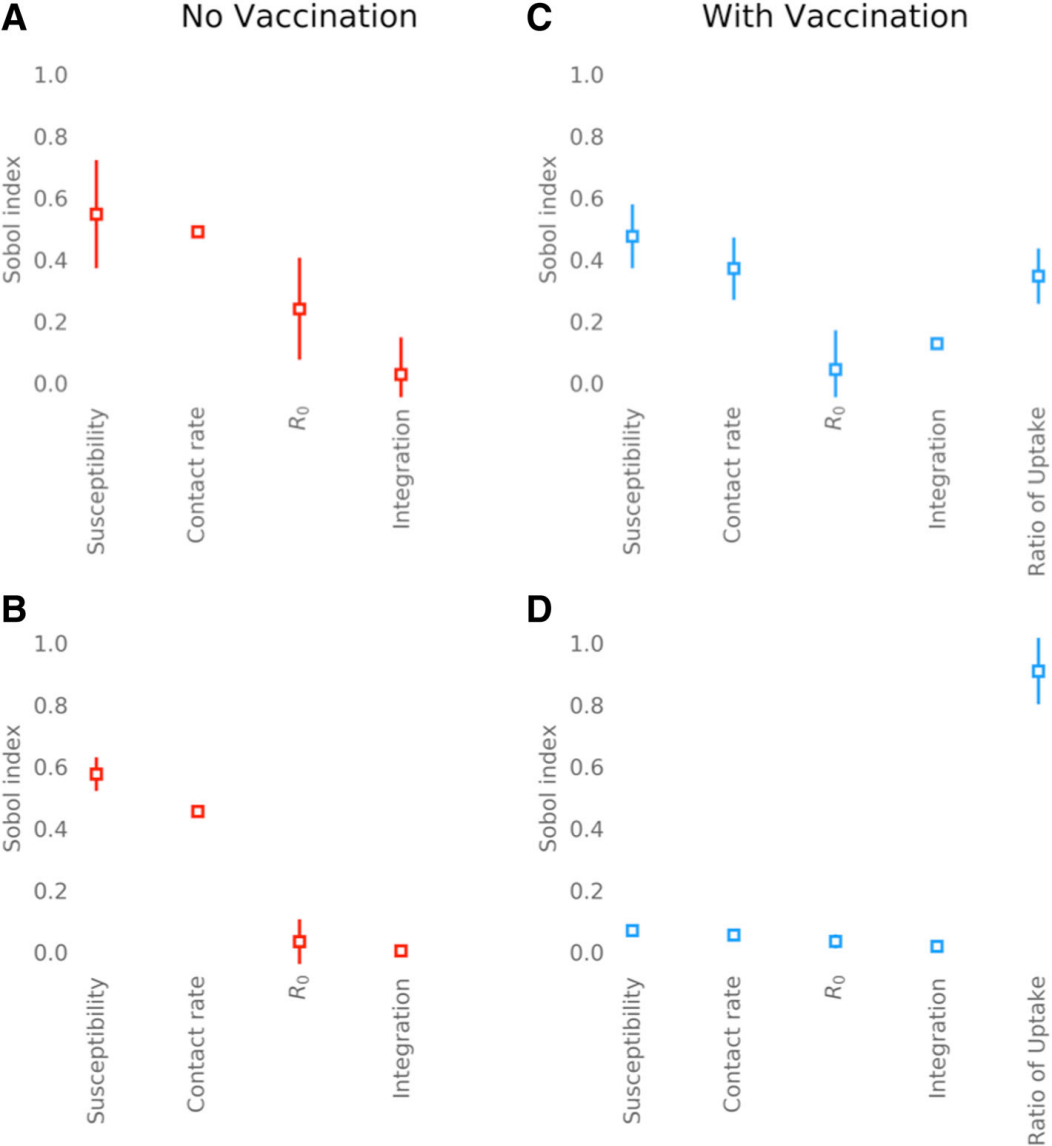
Total Sobol′ indices, *S*_*T*_, for contact (*χ*), susceptibility (*η*), integration (*ξ*), infectivity (*R*_0_) and difference in vaccination coverage (*V*_*H*_/*V*_*L*_) relative risks for rubella and influenza. **a** Influenza in the elderly (60+ years) with no vaccination. **b** Rubella in women of childbearing age (15–45 years) with no vaccination. **c** Influenza in the elderly with vaccination coverage at 37% (80% of the critical vaccination threshold). **d** Rubella in women of childbearing age (15–45 years) with vaccination coverage at 67% (80% of the critical vaccination threshold). Error bars show 95% confidence interval

Without vaccination the magnitude of the inequality (i.e. relative risk of infection for the high-transmission group) in influenza was most sensitive to the relative susceptibility of the social groups (*S*_T_ = 0.55) and their relative contact rate (*S*_T_ = 0.48) (Fig. 6a). The same was true for rubella (for relative susceptibility *S*_T_ = 0.58; for relative within-group contact rate *S*_T_ = 0.46). By comparison, sensitivity to integration between the two groups was relatively small, however greater for influenza than rubella (*S*_T_ = 0.03 vs. 0.004, respectively) (Fig. 6b).

#### Vaccination era

Additional variance introduced by differences in vaccine uptake between social groups caused a reduction in the relative sensitivity of inequalities to all other parameters, with the exception of integration. Nonetheless, for influenza, inequality in the disease risk between the two social groups remained most sensitive to relative susceptibility and contact rate (*S*_T_ = 0.48; *S*_T_ = 0.37). In contrast, inequalities were relatively insensitive to relative vaccine uptake (*S*_T_ = 0.35) (Fig. 6c). Sensitivity to the integration between social groups also increased relative to no vaccination (*S*_T_ = 0.13). For rubella, relative vaccine uptake between the two social groups had the greatest influence on inequality (*S*_T_ = 0.91), diminishing the relative sensitivity of inequality to relative susceptibility and contact rate of the social groups such that they were negligible (Fig. 6d).

## Discussion

Differences in incidence of infectious diseases between social groups have been observed; however, the factors that drive these inequalities are not well quantified. Moreover, the impact of vaccination on these inequalities is unclear. We developed a novel mathematical model to simulate influenza and rubella in two connected social groups and assessed the role of differences in two key factors—contact rate and susceptibility—on inequalities as well as the impact of vaccination. Our model suggested that these factors could be responsible for substantial differences in disease epidemiology between social groups. Therefore, these factors may play a significant role in driving observed inequalities in infectious disease outcomes. Furthermore, the results suggest that the impact of these factors on inequalities depends on the characteristics of the pathogen, as we show that the same differences in transmission are likely to cause greater inequality in influenza than rubella. Vaccination can exacerbate the inequalities even when the uptake is equal between the groups.

These observations have four important implications for public health and immunisation strategies. First, inequality in health is an area of high importance amongst public health authorities [5, 24]. As such, there is an appetite for policy that avoids and reduces inequalities in infectious disease outcome [25, 26]. To this end, effort is spent attempting to provide equal distribution of vaccination across social groups in the population [27]. However, our results indicate that equal vaccination uptake could, paradoxically, increase inequalities into high-transmission groups, if the vaccine coverage is not high enough to eliminate disease. This result indicates that equal vaccination is not an appropriate measure of equitable intervention, and inequality in disease burden must be evaluated directly.

Second, groups who have social characteristics that place them at a higher risk of infection and who also have a reduced vaccination uptake may be vulnerable to amplified inequalities. For example, during pH1N1 in 2009, Black and Hispanic populations had a lower uptake of influenza vaccination than the White population in the USA [28]. In addition, countries with self-financed or partially self-paid vaccination programmes may discourage more materially deprived groups from vaccinating; studies [29, 30] in Poland and South Korea have identified that lower uptake of vaccination correlates with low socio-economic status. This leaves the possibility that low uptake may correlate with factors contributing to transmission.

Third, the factors that most influence inequality depend on the underlying disease dynamics; therefore, intervention efforts must be disease- and population-specific. For example, our results indicate that differences in vaccine uptake are more important in creating inequalities in rubella than differences in factors associated with transmission rate. This is reflected in the small (0.3–2.3%) change from equal vaccine uptake required to mitigate differences in contact rate or susceptibility (Fig. 4). However, inequalities in influenza are more sensitive to differences in transmission-related factors than differences in vaccine uptake. This contrast was evidenced when low vaccine uptake in more affluent social groups created ’a reversal of health inequalities’ with higher prevalence in more affluent areas during a measles outbreak in London, UK in 2001–2002 [31]. In contrast, the same geographical region saw a higher attack rate of pH1N1 in more deprived areas [9] only 7 years later. This finding suggests that, notwithstanding the potential to increase existing inequalities, for diseases like rubella, equal vaccine uptake may be the most practicable target for minimising post-vaccination inequalities in disease burden. However, the same approach may not be optimal for influenza.

Finally, we identified that inequalities resulting from differences in transmission are highly sensitive to the level of integration of sub-groups. The importance of integration between social groups becomes more pronounced for diseases with sub-optimal vaccine uptake. This result suggests that inequalities driven by differences in transmission rate or a difference in vaccine uptake may be more likely to occur in highly segregated populations. Our finding could explain inequalities in incidence of infectious disease in urban centres, where there is geographical clustering of social and ethnic groups. For example, central Birmingham, UK, which was heavily affected by pH1N1 in 2009, is an area where up to 80% of the population is South Asian, an ethnic group associated with higher risk of transmission [8]. This phenomenon may also contribute to increased risk of outbreaks of measles, often observed in isolated communities with low vaccination coverage [32, 33]. Our findings reinforce the notion that communities that are more isolated should be of particular focus when considering public health strategies for infectious disease. Further, our results highlight the importance of understanding the role of transmission-related factors in inequality in populations where social and ethnic groups are becoming more segregated, as inequalities could be set to increase [34].

Our influenza model predicts a relative risk of infection in an unvaccinated population of up to 2.05, within feasible values of social group mixing and susceptibility. This is broadly consistent with data from the pH1N1 epidemic in 2009. For example, a case-control study from Ontario, Canada shows that East/Southeast Asian, South Asian and Black ethnicities had a significantly increased risk of acquiring pH1N1 relative to White Canadians (OR 1.33–4.50) [17]. Similarly, in New Zealand a seroprevalence study showed that Pacific Island populations were twice as likely to be infected during the 2009 pandemic than those of European ethnic identity [18]. Whilst there are many examples of observed inequalities in influenza [8, 9, 35–39], studies of inequalities associated with rubella and other endemic childhood infections are often focused on disparities in vaccine uptake rather than disease outcome [40].

Whilst much attention has been given to investigating the impact of transmission heterogeneity on the overall effectiveness of control strategies [41, 42], we build on this work by considering the role of heterogeneity in influencing inequalities in infectious disease outcomes, rather than the overall disease burden. Transmission models have previously been developed to evaluate the impact of social structure on observed inequalities in reported incidence of pandemic and seasonal influenza [43, 44]. By using socio-economic census data, these studies can replicate some of the location-specific inequalities between pre-defined social groups. However, because it is difficult to disentangle the drivers of inequality underlying these socio-economic groups, the models do not provide a fully generalisable framework in which to evaluate inequality. To overcome this issue, we developed a ‘bottom up’ approach, in which potential transmission-related drivers of inequality are isolated and evaluated. By parameterising our model with empirical social mixing data, we can explicitly capture the contact patterns between age and social groups and the effect of vaccination. Our generalised framework therefore allows us to disentangle the relative impact of different drivers of inequality and the impact of vaccination on this inequality.

To enable a mechanistic understanding of the drivers of inequality, we made some simplifying assumptions. We assumed that the two social groups in our model have identical age structures and birth rates. It has been shown that differences in age structure and other demographic differences such as birth rate can also result in changes in transmission which lead to inequalities in incidence [43, 44] and the effectiveness of vaccination [45]. To remain consistent with this assumption, we corrected for age distribution when we calculated the range of differences in contact rate between the groups. Furthermore, we assumed gender non-specific contact patterns. In some settings gender differences may exist, particularly in rates of contact between adults and infants [46, 47]. Whilst this gender difference may also differ between social groups, a recent survey suggests that contact rates between mothers and children are broadly consistent across ethnic and socio-economic groups [47]. Our approach is general and aims to establish the relative impact of various drivers of inequality. As such, our results should not be considered as indicative of the magnitude of specific inequalities, but rather as the potential for difference in transmission to explain inequalities and the qualitative nature of the inequalities that may arise from such drivers. We hope the results can be used to target additional analyses at specific scenarios where differences in transmission may arise, for example, where differences in household size distribution or high levels of segregation between social groups prevail.

## Conclusion

Differences in contact behaviour and susceptibility to infection could cause substantial inequality in infectious disease-related health outcomes, particularly those related to influenza outbreaks or infections with similar epidemiology. Such inequalities have a highly non-linear relationship with vaccination, which is sensitive to the underlying epidemiology of the infection, ultimately resulting in an increase in inequality after sub-optimal vaccination, even when uptake is equal across the entire population. As such, we advocate measurement of health outcomes rather than vaccination coverage when quantifying the equality of protection across multiple social groups. Moreover, targeted vaccination in known risk groups may reduce overall inequalities in the case of influenza outbreaks. However, due to the high sensitivity of rubella inequalities to differences in vaccination coverage, this is not a recommended course of action in this case or for similar infections.

## Additional file

Additional file 1: Supporting methods, additional results and sensitivity analyses. (DOCX 1495 kb)

## Abbreviations

CVT: Critical vaccination threshold
pH1N1: Pandemic influenza A H1N1 (2009)
WCA: Women of childbearing age
