# Supplementary material for: Quantifying the impact of social groups and vaccination on inequalities in infectious diseases using a mathematical model
Source: BMC Med. 2018 Sep 26;16:162. doi: 10.1186/s12916-018-1152-1 (PMC6156851; doi:10.1186/s12916-018-1152-1)
Supplement: Supplementary file 1 — Supporting methods, additional results and sensitivity analyses. (DOCX 1495 kb) [file 12916_2018_1152_MOESM1_ESM.docx]

**Additional file 1:** Quantifying the impact of social groups and vaccination on inequalities in infectious diseases using a mathematical model

**Contents:**

- ***Mathematical model***
  - ***Model Structure***
  - ***Calculating force of infection***
- ***Parameterisation of the model***
  - ***Integration of Social-groups***
  - ***Difference in rate of contact between groups***
- ***Epidemiological results over the full parameter ranges***
- ***Sensitivity analyses***

***Mathematical model***

*Model Structure*

We developed a Susceptible Exposed Infected Recovered (SEIR) model with two social groups and 15 age groups. The full system of ordinary differential equations is expressed as:

$$D\left( C_{i,G} \right)= \left\{ \begin{matrix} \left( 1-\rho\right)\mu-C_{i,G}a_{i} & ; & i=1, C=S & & \\ \rho\mu-C_{i,G}a_{i} & ; & i=1, C=V & & \\ -C_{i,G}a_{i} & ; & i=1, C\neq S,V & ; & C_{i,G}\in\left\{ S_{i,G}, E_{i,G},I_{i,G},R_{i,G},V_{i.G} \right\} \\ C_{i-1,G}a_{i-1}-C_{i,G}a_{i} & ; & 1<i<n & & \\ C_{i-1,G}a_{i-1}-\frac{C_{j,G}\mu}{p_{i,G}} & ; & i=n & & \end{matrix} \right.$$

$$p_{i,G}=S_{i,G}+E_{i,G}+I_{i,G}+R_{i,G}+V_{i.G}$$

$$\lambda_{i,H}=\sum_{j=1}^{n_{age}} \beta_{ij}\left( I_{j,A}+{\xi I}_{j,B} \right)$$

$$\lambda_{i,L}=\sum_{j=1}^{n_{age}} {\eta\beta}_{ij}\left( {\xi I}_{j,A}+{\chi I}_{j,B} \right)$$

$$\frac{dS_{i,G}}{dt}=-\sum_{j=1}^{n} \lambda_{i,G}S_{i,G}-\mu S_{i,G}+D\left( S_{i,G} \right)$$

$$\frac{dE_{i,G}}{dt}=\sum_{j=1}^{n} \lambda_{i,G}S_{i,G}-\sigma E_{i,G}+D\left( E_{i,G} \right)$$

$$\frac{dI_{i,G}}{dt}=\sigma I_{i,G}-rI_{i,G}+D\left( I_{i,G} \right)$$

$$\frac{dR_{i,G}}{dt}=\kappa R_{i,G}+D\left( R_{i,G} \right)$$

$$\frac{dV_{i,G}}{dt}=D\left( V_{i,G} \right),$$

where $S_{i,G}$*,* $E_{i,G}$*,* $I_{i,G}$*,* $R_{i,G}$*,* $V_{i.G}$ are the proportion of the population in age group *i* and in social group *G* that are susceptible, exposed (infected but not infectious), infectious, recovered and vaccinated respectively. We define the parameters:

*μ* is birth and death rate

$\rho$ is the proportion of the population vaccinated

$\beta_{ij}$ is the rate of transmission from age group *j* to age group *i*

$\eta$ is the relative susceptibility of group *L*

$\chi$ is the relative contact rate of group *L*

$\xi$ is the rate of contact between social groups relative to within group *H*

$p_{i,G}$ is the proportion of the population in age group *i* and social group *G* \in {*H*, *L*}

$\sigma$ is the rate at which individuals become infectious after being infected

$\kappa$ is the rate at which individuals recover from infection (cease to be infectious)

$a_{i}$ is the rate at which the population moves from age group *i* to age group *i*+1

*Calculating the force of infection,* $\lambda$

To ensure that all parameterisations of differences in contact, susceptibility and social integration result comparable epidemiology, we kept the basic reproduction number ($R_{0}$) constant by scaling the next generation matrix **R** linearly such that its largest eigenvalue was equal to the correct value of *R*_0_**.**

Each element of the next generation matrix, $R_{ab}$, gives the expected number of cases in age and social group *a* resulting in transmission from a single case in age and social group *b* in an otherwise totally susceptible population. The force of infection vector can be written in terms of the next generation matrix R, which is a function of the matrix of transmission parameters, $\mathbf{B}$ and the infectious period $\gamma$.

$$\vec{\lambda}=\mathbf{B}I=\frac{1}{\gamma}\mathbf{R}I$$

Neglecting age groups initially, we rewrite the force of infection as a function of the transmission rate within age group H, $\beta$ and the social interaction between the two social groups, **X**:

$$\vec{\lambda}=\beta\mathbf{X}I,$$

where,

$$\left( \begin{matrix} \lambda_{H} \\ \lambda_{L} \end{matrix} \right)=\beta\left( \begin{matrix} 1 & \xi\\ \eta\xi& \eta\chi\end{matrix} \right)\left( \begin{matrix} I_{H} \\ I_{L} \end{matrix} \right)$$

We introduced age groups by first defining **P** as a normalised age dependent social contact matrix (such that the elements sum to unity) where each element, $P_{ij},$ is the rate of contact between age group *j* and age group *i* per individual in group *i.* We construct a matrix, $\mathbf{P}_{\mathbf{total}}\boldsymbol{,}$ to account for the transmission between age groups and between two social groups:

$$\mathbf{P}_{\mathbf{total}}=\left( \begin{matrix} \mathbf{P} & \mathbf{P} \\ \mathbf{P} & \mathbf{P} \end{matrix} \right)$$

.

We take the Hadamard (element-by-element) product of $\mathbf{X}$ and $\mathbf{P}_{\mathbf{total}}$ to give a normalised age and social group dependent next generation matrix, $\mathbf{R}_{norm}$**.**

$$\mathbf{R}_{norm}=X\circ\mathbf{P}_{\mathbf{total}}\boldsymbol{=}\left( \begin{matrix} 1 & \xi\\ \eta\xi& \eta\chi\end{matrix} \right)\circ\left( \begin{matrix} \mathbf{P} & \mathbf{P} \\ \mathbf{P} & \mathbf{P} \end{matrix} \right)=\left( \begin{matrix} \mathbf{P} & \xi\mathbf{P} \\ \eta\xi\mathbf{P} & \eta\chi\mathbf{P} \end{matrix} \right)$$

The next generation matrix is proportional to this normalised matrix,

$$\mathbf{R}=r\mathbf{R}_{norm}$$

We select a value of *r* to give a fixed $R_{0}$, defined as the spectral radius of$\mathbf{R}$**.**

An alternative approach is to vary the ratio of total contact rate in group L and group H by adapting the parameterisation to the following form.

$$\mathbf{R}_{norm}=X\circ\mathbf{P}_{\mathbf{total}}\boldsymbol{=}\left( \begin{matrix} 1 & \xi\\ \eta\xi& \eta\chi' \end{matrix} \right)\circ\left( \begin{matrix} \mathbf{P} & \mathbf{P} \\ \mathbf{P} & \mathbf{P} \end{matrix} \right)=\left( \begin{matrix} \mathbf{P} & \xi\mathbf{P} \\ \eta\xi\mathbf{P} & \eta\chi'\mathbf{P} \end{matrix} \right)$$

Where, $\chi^{'}=\chi\left( \chi-1 \right)\xi$. The two approaches were found to have consistent results (results not shown).

***Parameterisation of the model***

***Integration of Social-groups***

To estimate the proportion of contacts occurring between social groups, we use data collected as part of the Great Britain arm of the POLYMOD survey[1]. Participants were asked to complete a diary of social contacts made over a 24-hour period. As part of the survey, participants recorded the location of each contact event (home, work, school, leisure, transport or other place) and the duration of each contact. For each participant we assume that contact events that occurred within their home were with a member of their own social group. The mean number and time spent with household contacts account for 43% of contact events and for 47% of the total duration of contact the participants report, respectively. In addition, we assume 70–90% of contact that occurred outside the participant’s home was also with members of the participant’s own social group. The non-household contacts in the same social group then accounted for a mean of 44–56% of contact events and 37–48% of total duration of contact. We assume all remaining 10– 30% of non-household contacts belong to the other social group. To calculate the value of the integration parameter, $\xi,$we use the ratio between the contact with other social groups and with the participant’s social group for each participant in the data set. As the mean ratio was between 0.06 and 0.22 for number of contacts and 0.06 and 0.20 for total duration of contacts (Figure 2), we set $\xi$ between 0.05 and 0.25.

**B**

**A**


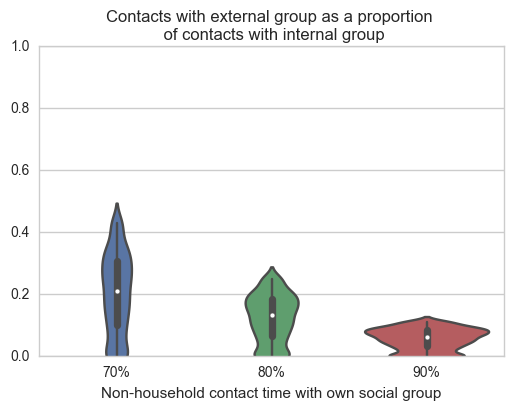

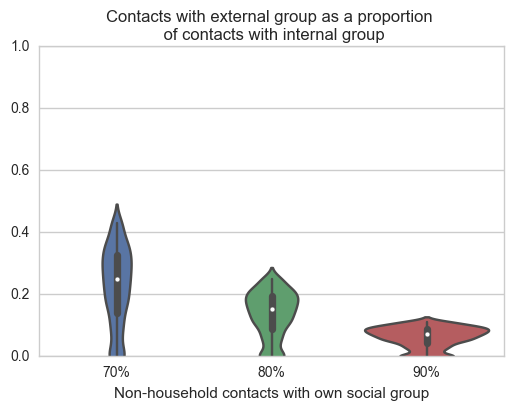


**Figure S1** Distribution of the ratio of A) number and B) total duration of contacts within the participants social group and outside of the participants social group (using the GB arm of the POLYMOD contact survey data).

***Difference in rate of contact between groups***

We also estimated an appropriate range for the difference in rate of contact between groups by also analysing data collected as part of the POLYMOD survey. Firstly, we calculated the number of contacts from the same social group, under the same definition as for integration of social groups.

As part of the POLYMOD survey household size of the participants was recorded. In this data and other contact surveys, household size has been shown to be a good predictor of contact rate, with higher rates in members of larger households[1–7]. In addition, household size distribution can vary significantly between social and ethnic groups. In order to choose an appropriate range for the difference in rate of contact between groups, we created 5 subsets of the sample population by sorting by Household size. We first stratified the population into 16 5-year age groups. To ensure the age distribution of each subset remained the same, subsequently we stratified each age group into 5 quintiles based on household size. We then assemble five final quintiles that comprise similar age distributions (Figure 2) but differing household size distributions (Figure 3). Consequently, the distribution of number of contacts and total duration of contact is also different for each of the quintiles (Figure 4 and Figure 5).

We calculate the ratio of mean number of contacts for each pair of quintiles to give a range of values for the relative rates of contact between two social groups (Figure 7). We use this range as our relative difference in within-group contact parameter ($\chi$ = 0.65 – 0.95).


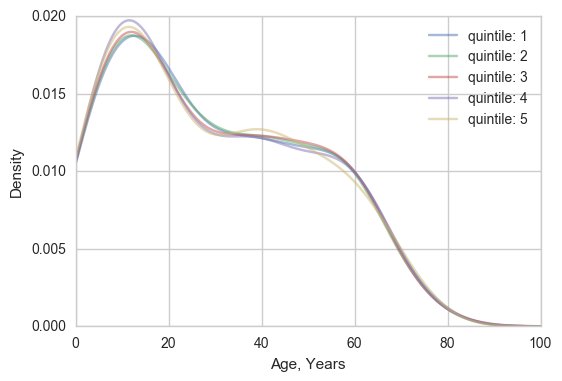


**Figure S2** The age distribution of each household size quintile


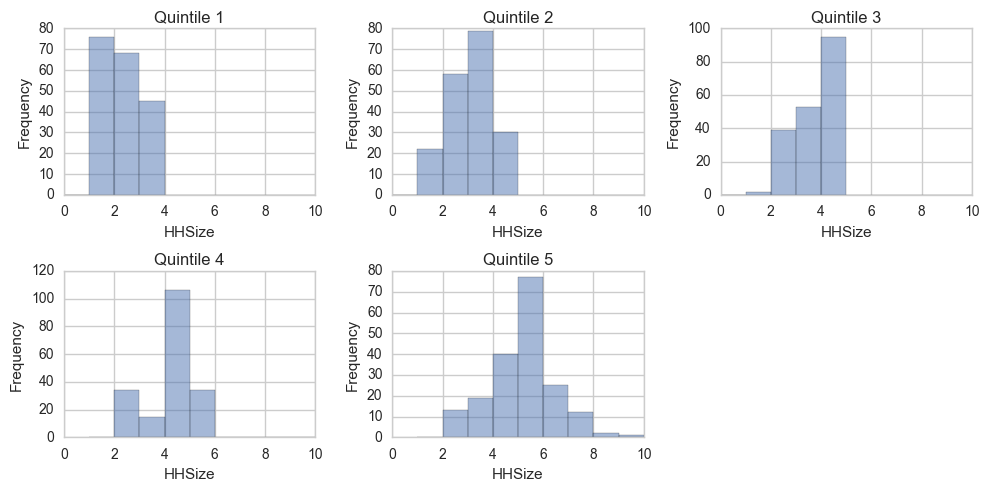


**Figure S3** Household size distributions of quintiles across all ages


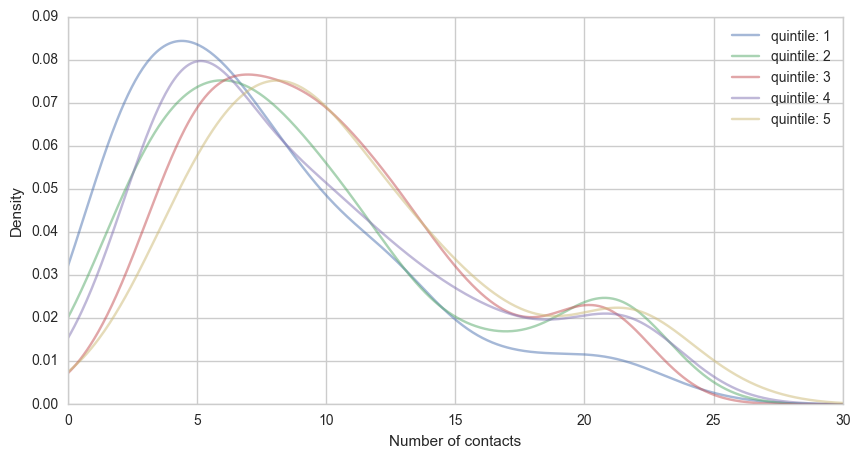


**Figure S4** Kernel density estimate showing the distribution of number of contacts in each quintile


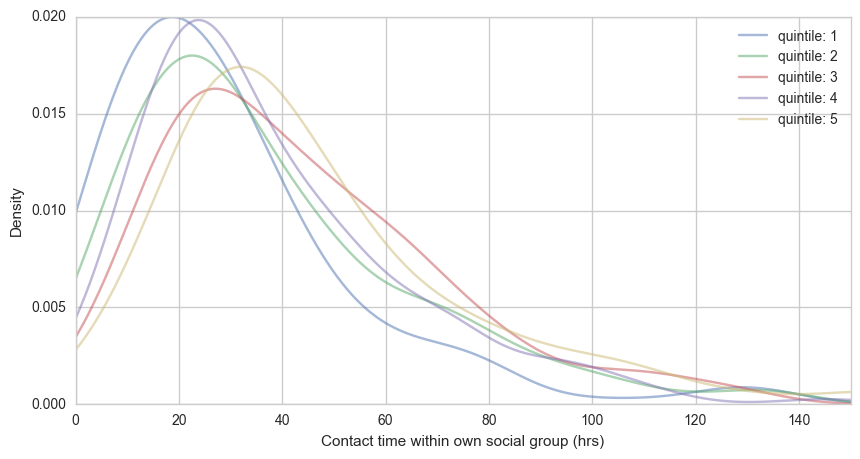


**Figure S5** Kernel density estimate showing the distribution of total duration of contacts in each quintile

**B**

**A**


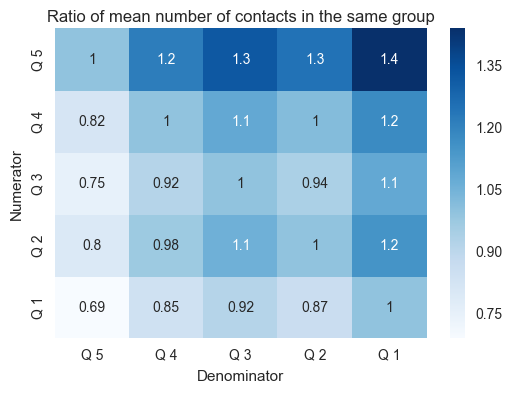

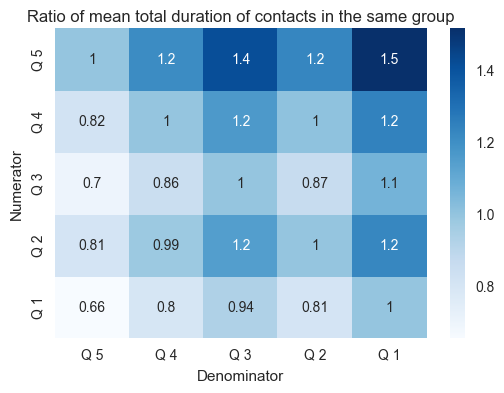


**Figure S6** Colour maps showing the ratio of A) number of contacts and B) total duration of contacts between each of the quintiles of POLYMOD participants stratified by household size. The range of the ratios was subsequently used to inform the contact rate in group L relative to group H.

***Epidemiological results over the full parameter ranges***

To measure the relative risk of infection resulting from a range of differences in contact rate, susceptibility and integration of social groups, we model an outbreak of influenza and endemic rubella in two social groups. We alter relative contact rates within social groups by varying a parameter, $\chi$, relative susceptibility in the social groups by varying a second parameter, $\eta$, and the level of integration of the two social groups with each other by varying another parameter, $\xi$. Figures in the main text only contain results for $\xi=0.15$. This section presents figures for results with different values of $\xi.$

Figure 7 to Figure 12 show the cumulative incidence of influenza over an outbreak and cumulative risk of infection for rubella over the first 75 years of life. Each shows the result with no difference between the social groups ($\chi, \eta=1)$and the range of results in each social group over the full range of either relative contact rate, $\chi=0.65-0.95$ (Figure 7 – Figure 9), or relative susceptibility, $\eta=0.65-0.95$ (Figure 10 – Figure 12), with for a fixed value of $\xi\in\{0.05, 0.15, 0.25\}$.

Adding differences in contact rate and susceptibility between the two social groups changes the epidemiology and the risk of infection: specifically the risk of infection with influenza increases in group H and reduces in group L. When we increase the integration between the social groups ($\xi$), there is a reduction in the change in risk relative to when the subgroups are identical. When vaccination is introduced into the model at 80% of the critical vaccination threshold, The epidemiology of both infections changed markedly with overall reduction of risk of infection in both groups. However, indirect protection is greater in the social group L; the with lower rates of transmission (contact rate or susceptibility). This difference in a greater reduction in disease risk than in group H. The consequence is an increase in the relative risk of infection in influenza in group H and a greater risk of infection from rubella in group H than group L (Figure 13).


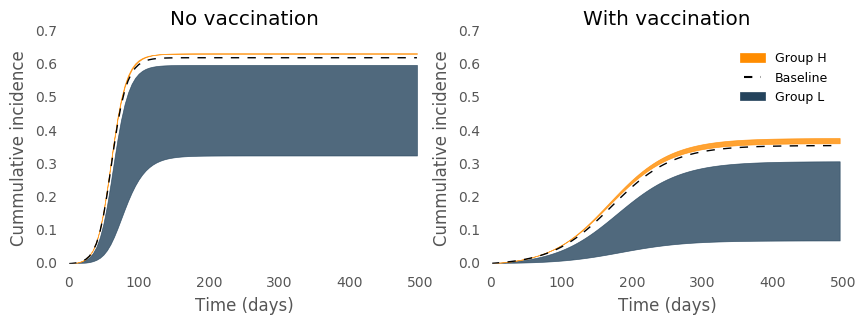


**D**

**B**

**C**

**A**


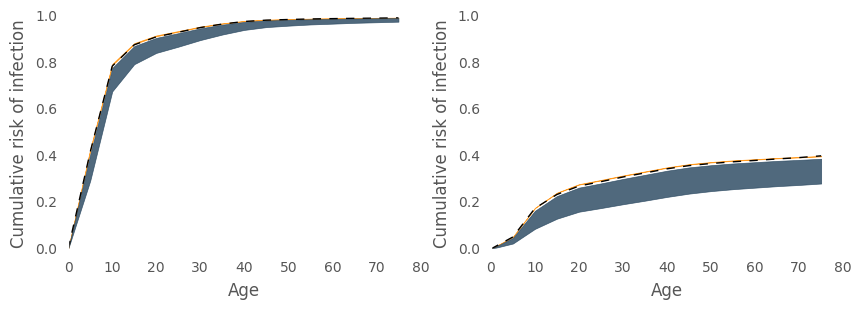


**Figure S7** The change in infection over time or by age predicted by the model for seasonal influenza and rubella. With no differences between two population groups under baseline parameters shown in black dashed line and with differences in susceptibility and contact rate for group H in the orange region and group L in the navy region. The results are based on a ratio of contact rate between groups ($\chi=0.65$ – $0.95)$ and low integration ($\xi=0.05$) . A) the cumulative incidence of influenza over a single outbreak with no vaccination, B) shows the proportion of population infected with Rubella by age at endemic equilibrium with no vaccination, C) the cumulative incidence of influenza in remaining unvaccinated individuals with 37% vaccine uptake (80% of the critical vaccination threshold) and D) the proportion of remaining unvaccinated population infected with Rubella by age with 67% vaccine uptake (80% of the critical vaccination threshold).


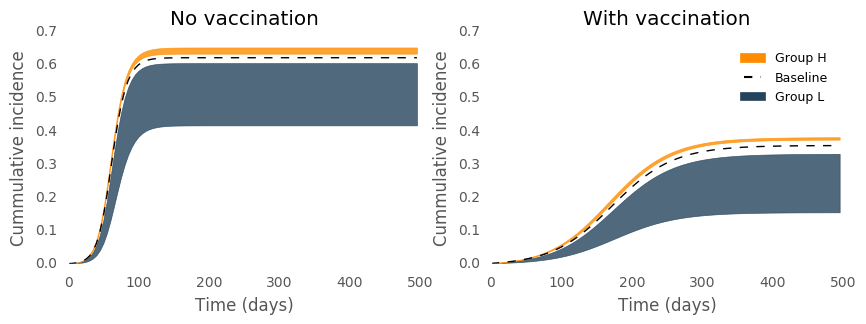


**D**

**B**

**C**

**A**


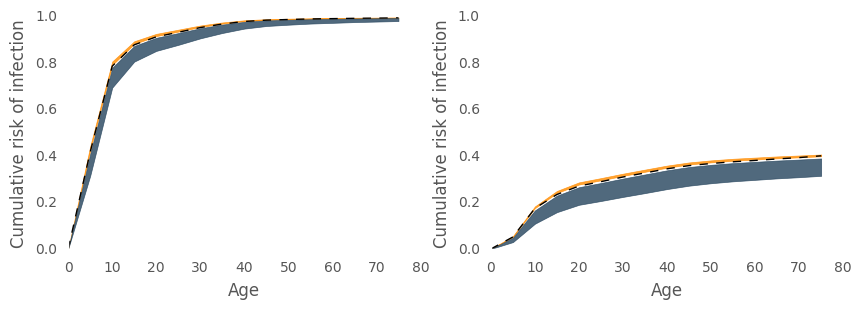


**Figure S8** As 7 but with intermediate integration ($\xi=0.15$)


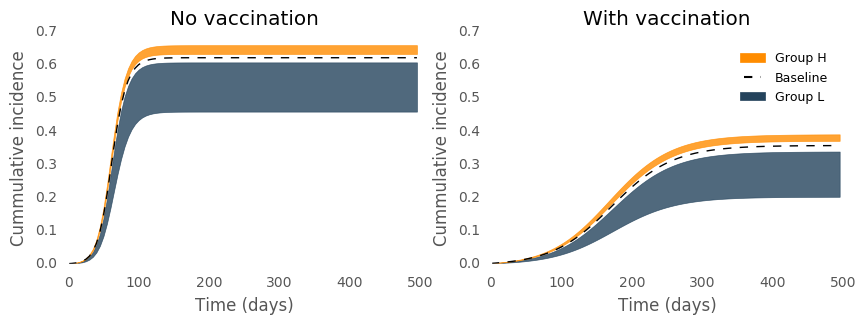


**D**

**B**

**C**

**A**

**
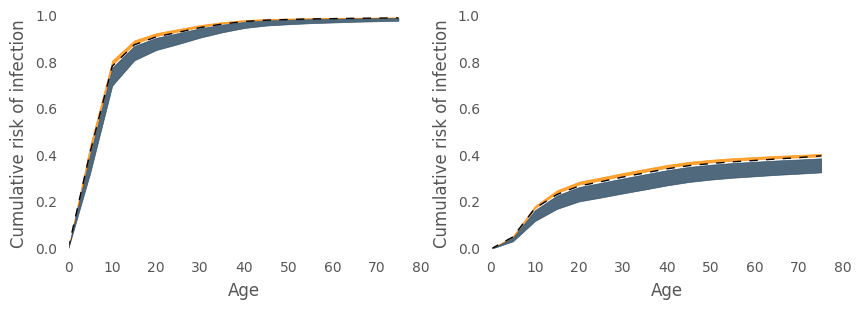
**

**Figure S9**  As 7 but with high integration ($\xi=0.25$)


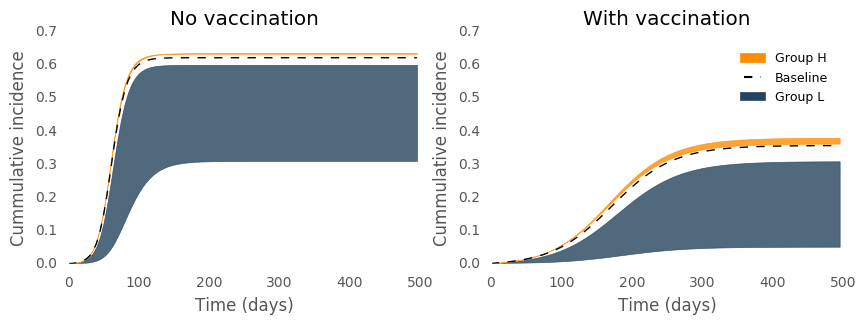


**D**

**B**

**C**

**A**


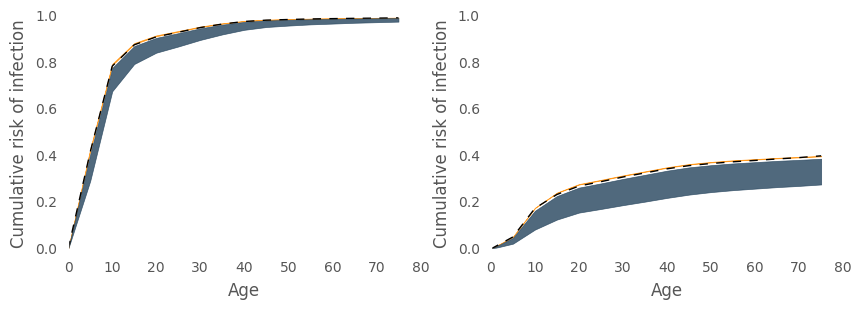


**Figure S10** The change in infection over time or by age predicted by the model for seasonal influenza and rubella. With no differences between two population groups under baseline parameters shown in black dashed line and with differences in susceptibility and contact rate for group H in the orange region and group L in the navy region. The results are based on a ratio of susceptibility between groups ($\eta=0.65$ – $0.95)$ and low integration ($\xi=0.05$). A) the cumulative incidence of influenza over a single outbreak with no vaccination, B) the proportion of population infected with Rubella by age at endemic equilibrium with no vaccination, C) the cumulative incidence of influenza in remaining unvaccinated individuals with 37% vaccine uptake (80% of the critical vaccination threshold) and D) the proportion of remaining unvaccinated population infected with Rubella by age with 67% vaccine uptake (80% of the critical vaccination threshold).


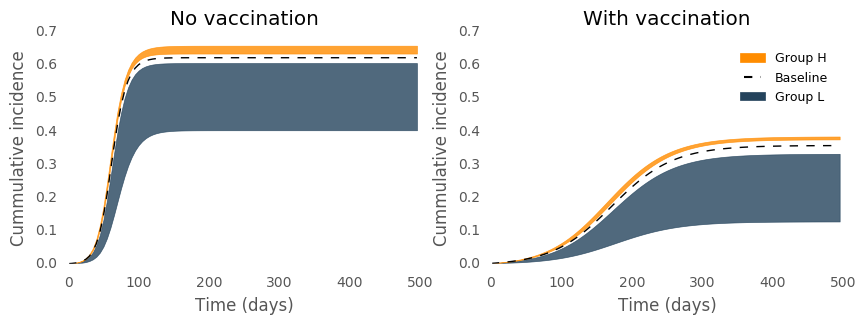


**A**

**C**

**B**

**D**


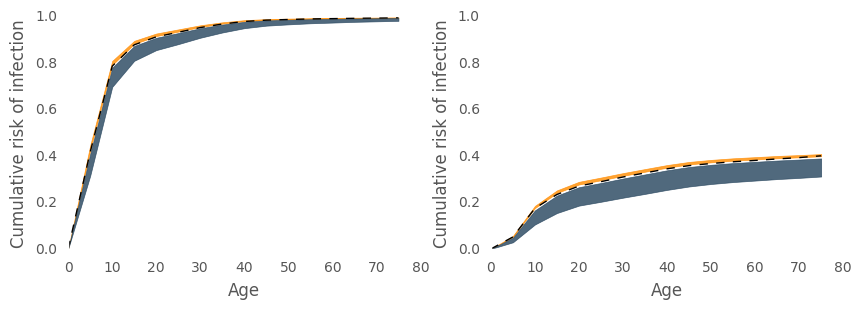


**Figure S11** As 10 but with intermediate integration ($\xi=0.15$)


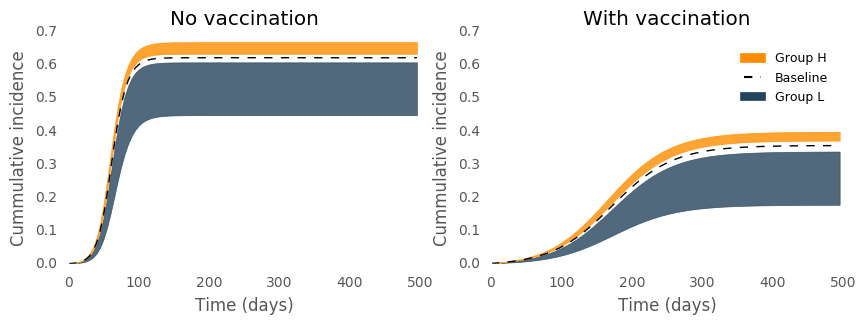


**D**

**B**

**C**

**A**


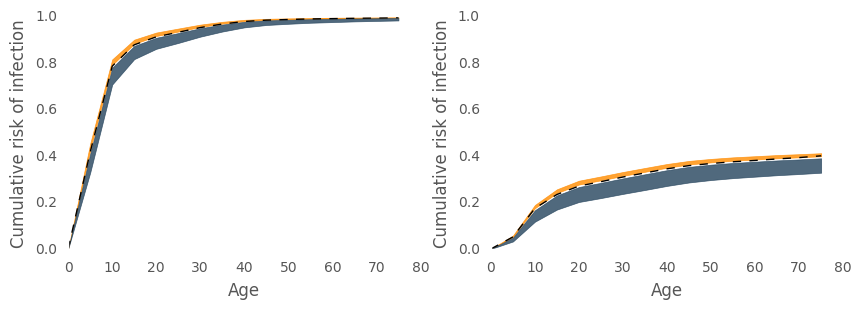


**Figure S12** As 10 but with high integration ($\xi=0.25$)


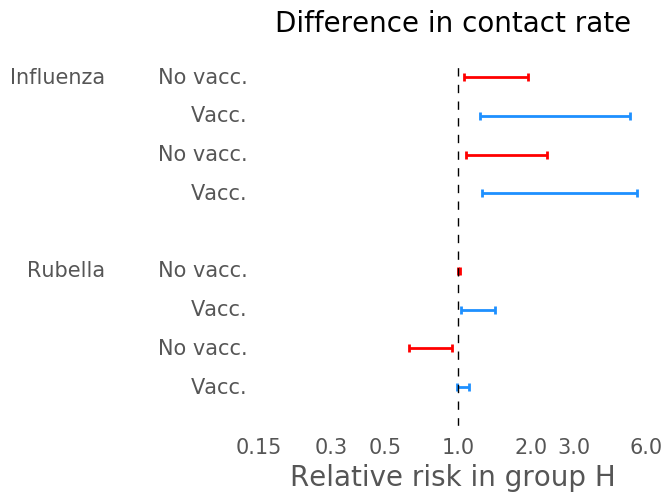

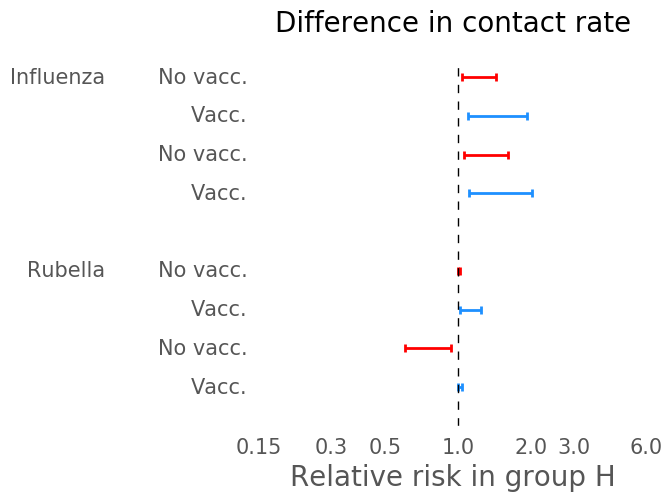


**A**

**C**


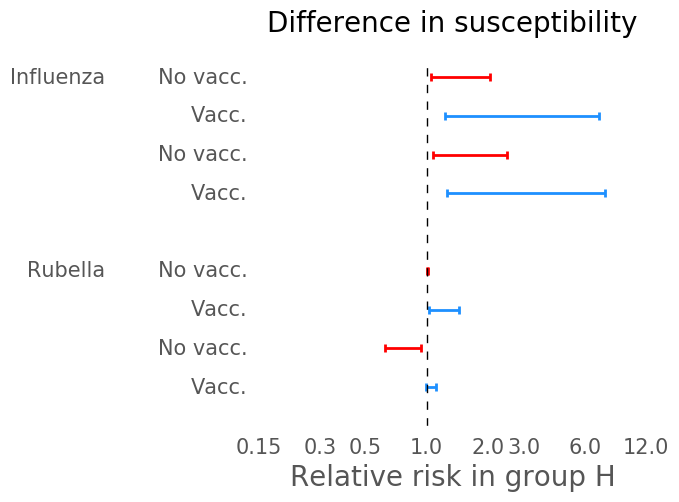

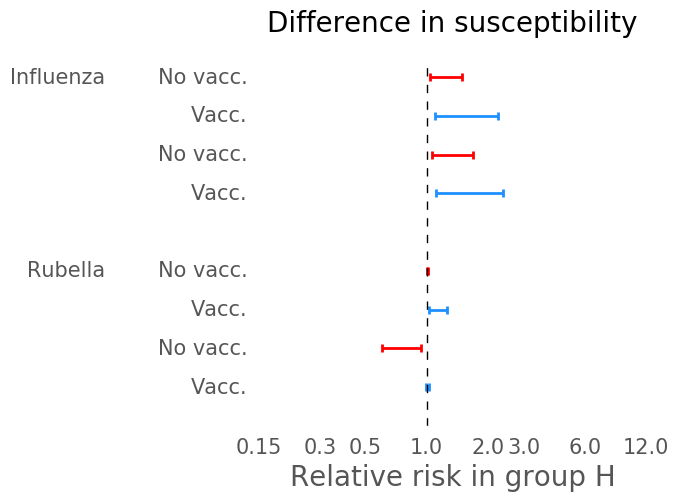


**D**

**B**

**Figure S13** Risk of infection in group H relative to group L in the total population and in risk groups, elderly and women of childbearing age (WCA). Relative risks shown with no vaccination and vaccination at 80% of critical vaccination threshold (37% for influenza and 67% for rubella). Forest plots show ranges of relative risk for a range of ratio of in contact rate in social groups ($\chi$=0.65-0.95) and ratio of susceptibility in social groups ($\eta$=0.65-0.95) with integration of $\xi$ = 0.05 (A) and B)) and integration of $\xi$ = 0.25 (C) and D)).

***Sensitivity analyses***

To test the sensitivity of our results to some of the key assumptions in our model, here we vary the relative size of the social groups and the age-specific mixing.

**Size of social groups**

The main analysis assumes that the size of each social group is equal (50% of the population each).. Now we assume 80% of the population in the low transmission group, group L, leaving 20% of the population in group H (and vice versa).

**Age assortativity in contact matrix**

To test the sensitivity of our findings to community structure, we vary relative age-stratified mixing pattern in between the two social groups. First we adjust the ‘age-assortativity’ (preference in contact with one’s own age group over other age groups). We adjusted this using an eigen decomposition method employed by Küchenhoff et al [8], which allows the relative strength of the ‘off-diagonal’ terms of the mixing matrix to be adjusted with single parameter *k*. When *k* > 1 age assortativity decreases (contact between age-groups increases), when *k* < 1 age assortativity increases (contact between age-groups decreases). Transmission matrices for *k* = 0.6 and *k* = 1.8 in Group L are shown in Figure 14.


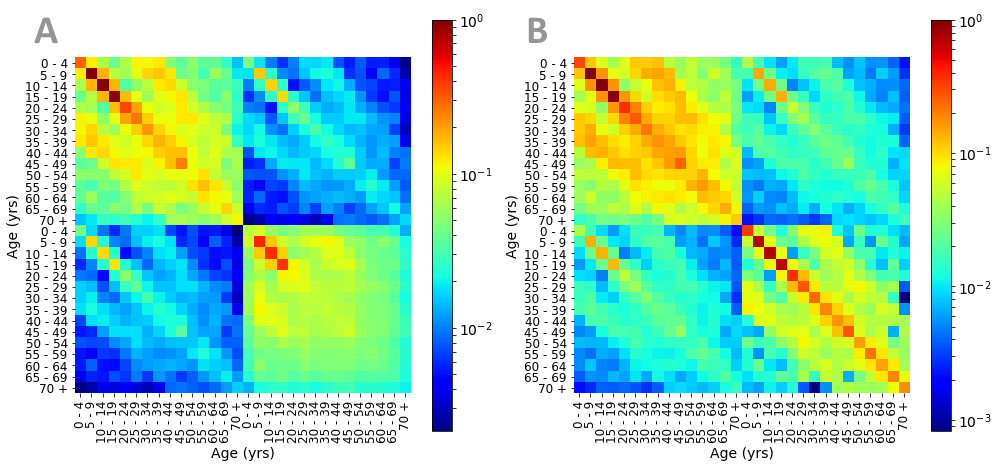


**Figure S14** Examples of the full, age and social group structured transmission matrix with A) less age assortativity of Group L (*k*=1.8) and B) more age assortativity in group L (*k*=0.6)

We find that changing the age assortativity made little difference to the inequalities we measured, with the exception of rubella in women of childbearing age. In this special case, an increased age assortativity leads to higher rate of contact between children, decreasing the average age at infection in group L. In addition, lower rates of contact between adults and children leads to lower rate of transmission between children and susceptible adults. These factors have the effect of reducing the risk in group L relative to group H with no vaccination. With vaccination, the risk in group L relative to group H reduces as observed in our main analysis.

Conversely, decreasing age assortativity leads to lower rate of contact between children, increasing the average age at infection in group L. Higher rates of contact between adults and children leads to higher rate of transmission between children and susceptible adults. These factors have the effect of increasing the risk in group L relative to group H with no vaccination. With vaccination, the risk in group L relative to group H reduced, as observed in our main analysis.

Although the values for relative risk of rubella infection in women of childbearing age vary notably from the main analysis as assortativity is varied, the qualitative result from our analysis does not change. Namely, ….

**Cultural differences in age-specific contact patterns**

Here we change the age-specific mixing between social groups by using two distinct empirical social mixing patterns for each social group. Specifically, we assume that group L has age-specific contact rates parameterised with data from the Italian arm of the POLYMOD survey, while group H has age-specific contact rates from the UK arm of the survey. Similar to the results of explicitly changing the age assortativity, we note some changes to relative risks of rubella infection in women of childbearing age. However, these changes did not impact the qualitative result of our analysis (Figure 15 and Figure 16).


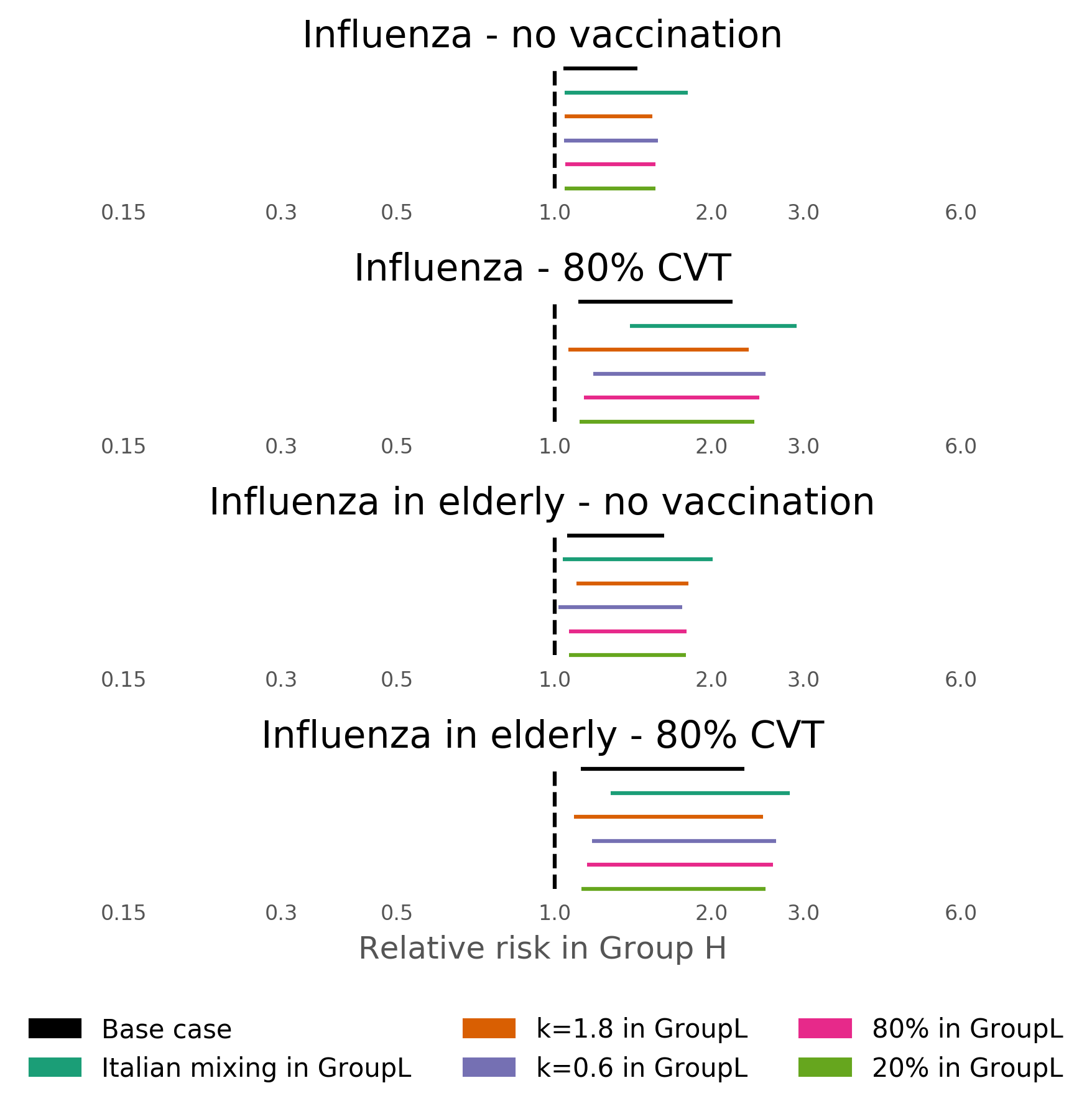


**Figure S15** Sensitivity analysis for relative risk in influenza infection due to difference in contact rate ($\chi$=0.65 – 0.95) with integration set as $\xi=0.15$.


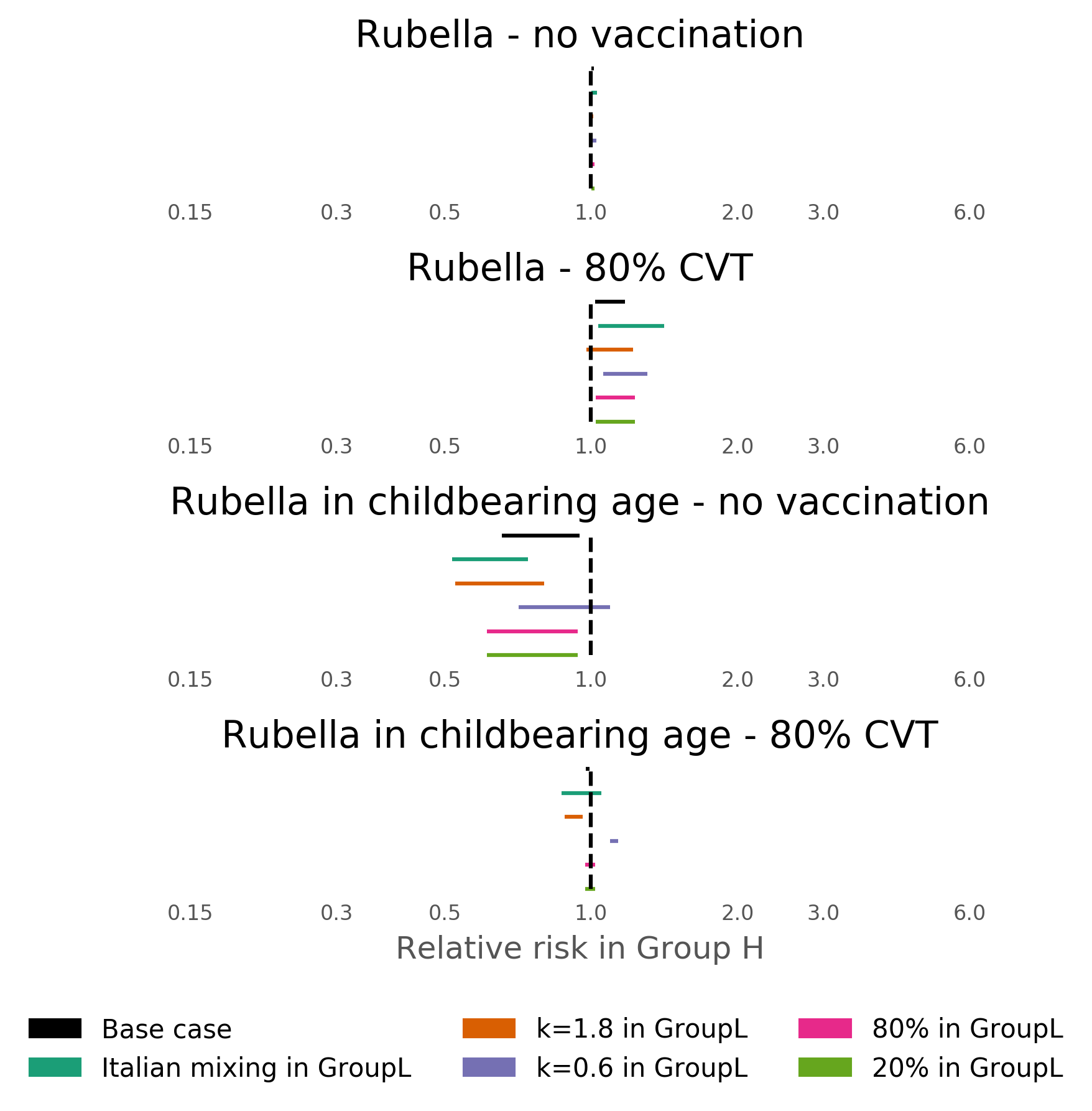


**Figure S16** Sensitivity analysis for relative risk in rubella infection due to difference in contact rate ($\chi$=0.65 – 0.95) with integration set as $\xi=0.15$.

**References**

1. Mossong J, Hens N, Jit M, Beutels P, Auranen K, Mikolajczyk R, et al. **Social contacts and mixing patterns relevant to the spread of infectious diseases.** *PLoS Med*. 2008, 5:e74.

2. Horby P, Pham QT, Hens N, Nguyen THTHTTY, Le QM, Dang DT, et al. **Social contact patterns in Vietnam and implications for the control of infectious diseases.** *PLoS One*. 2011, 6:e16965.

3. Stein ML, van der Heijden PGM, Buskens V, van Steenbergen JE, Bengtsson L, Koppeschaar CE, et al. **Tracking social contact networks with online respondent-driven detection: who recruits whom?**. *BMC Infect Dis*. 2015, 15:522.

4. Ibuka Y, Ohkusa Y, Sugawara T, Chapman GB, Yamin D, Atkins KE, et al. **Social contacts, vaccination decisions and influenza in Japan.** *J Epidemiol Community Health*. 2016, 70:162–7.

5. Read JM, Lessler J, Riley S, Wang S, Tan LJ, Kwok KO, et al. **Social mixing patterns in rural and urban areas of southern China.** *Proc Biol Sci*. 2014, 281:20140268.

6. Grijalva CG, Goeyvaerts N, Verastegui H, Edwards KM, Gil AI, Lanata CF, et al. **A household-based study of contact networks relevant for the spread of infectious diseases in the highlands of Peru.** *PLoS One*. 2015, 10:e0118457.

7. Béraud G, Kazmercziak S, Beutels P, Levy-Bruhl D, Lenne X, Mielcarek N, et al. **The French Connection: The First Large Population-Based Contact Survey in France Relevant for the Spread of Infectious Diseases.** *PLoS One*. 2015, 10:e0133203.

8. Küchenhoff H, Mwalili SM, Lesaffre E. **A General Method for Dealing with Misclassification in Regression: The Misclassification SIMEX**. *Biometrics*. 2006, 62:85–96.
